# A Rare Case of Severe Aortic Regurgitation Secondary to Tenting of Chordae Tendineae Strands: A Multimodality Imaging Approach for a Challenging Diagnosis

**DOI:** 10.3390/diagnostics15091071

**Published:** 2025-04-23

**Authors:** Dario Catapano, Santo Dellegrottaglie, Alessandra Scatteia, Carlo Maria Gallinoro, Carmine Emanuele Pascale, Luigi Falco, Emilio Di Lorenzo, Daniele Masarone

**Affiliations:** 1 Department of Translational Medical Sciences, University of Campania “Luigi Vanvitelli”, Via Leonardo Bianchi 1, 80131 Naples, Italy; 2 Advanced Cardiovascular Imaging Unit, Clinica Villa dei Fiori, 80011 Acerra, Italy; sandel74@hotmail.com (S.D.); a.scatteia@gmail.com (A.S.); carlomariagallinoro@gmail.com (C.M.G.); carmineemanuele.pascale@gmail.com (C.E.P.); 3 Department of Cardiology, AORN dei Colli Monaldi Hospital, 80131 Naples, Italy; luigifalco94@libero.it (L.F.); emilio.dilorenzo@ospedalideicolli.it (E.D.L.); danielemasarone@gmail.com (D.M.)

**Keywords:** aortic regurgitation, aortic chordae tendineae, multimodality imaging

## Abstract

We discuss a case of a patient who was referred to our department for an in-depth evaluation of aortic regurgitation severity and its underlying causes. By employing a multimodal imaging strategy that combined transthoracic echocardiography (TTE), transesophageal echocardiography (TEE), and cardiac magnetic resonance imaging (cMRI), we successfully identified a particularly rare cause of aortic regurgitation: chordae tendineae that lead to asymmetric retraction of the aortic cusps. Furthermore, this approach provided a clearer understanding of the aortic root anatomy and the hemodynamic effects of the regurgitant flow on the ventricle. This case demonstrates the diagnostic effectiveness of various imaging techniques and emphasizes the crucial importance of multimodal imaging for a thorough assessment of aortic valvular issues.

**Figure 1:**
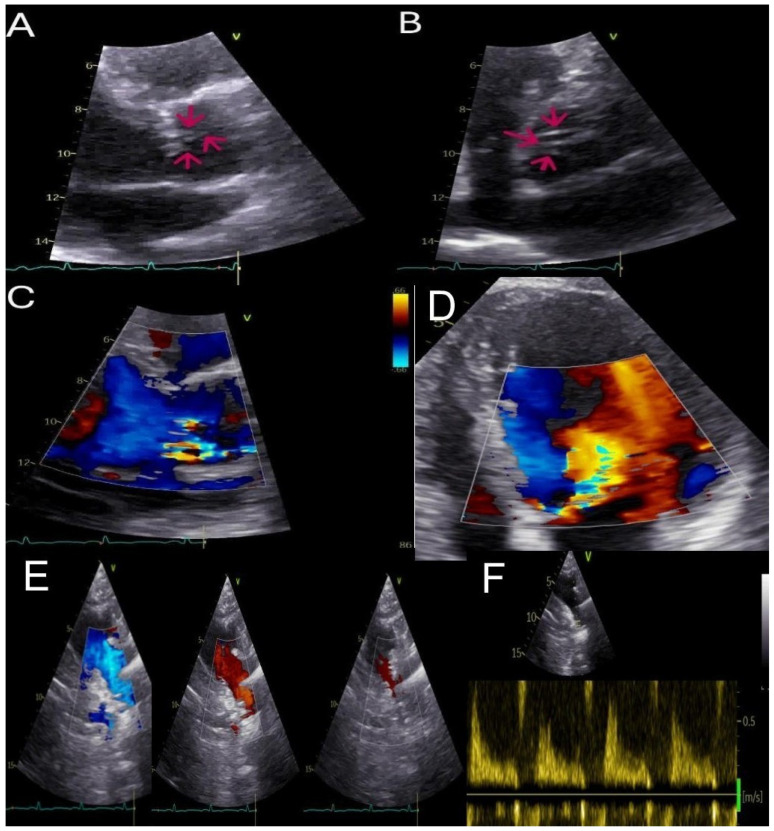
A 49-year-old male patient, overweight with a history of arterial hypertension and moderate aortic regurgitation with mild symptoms, was referred to our department for appropriate management. Trans-thoracic echocardiography (TTE) revealed moderate-to-severe aortic regurgitation following a multiparametric evaluation [1]: the aortic root (37 mm), annulus (26 mm), and sinotubular junction (30 mm) were of normal size, and the aortic valve was tricuspid, with normal systolic movement of the leaflets. During diastolic closure, subtle hyperechoic filaments of uncertain characterization were noted as shown by red arrows (PLAX 2D, panel (**A**); PSAX 2D, panel (**B**); Supplementary Video S1). Doppler evaluation demonstrated an eccentric regurgitant jet directed posteriorly, with a vena contracta (VC) of 5 mm (see, PLAX 2D color, panel (**C**)), which led to anterior mitral leaflet (AML) doming (see, apical 4CH view 2D color showing color aliasing of the aortic regurgitation jet along the entire anterior mitral leaflet, panel (**D**)). Although it was not possible to reliably measure the pressure half-time (PHT), other indicators of hemodynamic significance were identified. These included retrograde holodiastolic flow in the descending aorta with a peak end-diastolic reverse velocity of 0.26 m/s, as shown by color Doppler imaging (suprasternal 2D color view, from left to right proto-diastolic, mid-diastolic, tele-diastolic timeframes, panel (**E**); suprasternal 2D view with sample volume in the descending aorta, panel (**F**)), in addition to an increase in left ventricular end-diastolic volume despite preserved systolic function (end-diastolic volume indexed at 80 mL/m^2^, cardiac output 7.8 L/min, LVEF 55%).

**Figure 2:**
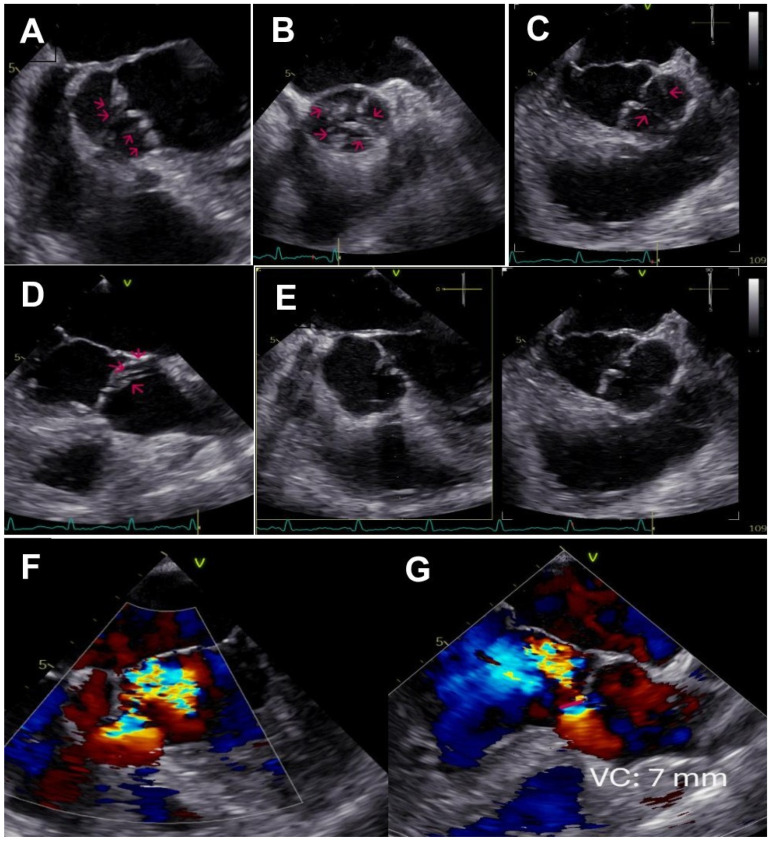
To better quantify the degree and the mechanisms of aortic regurgitation, the patient underwent transesophageal echocardiography (TEE), which revealed multiple chordae tendineae strands (ACTS) originating from the sinuses of Valsalva and inserting into the aortic cusps, as shown by red arrows in several mid-esophageal views (0°, panel (**A**); 45°, panel (**B**); 90°, panel (**C**); 113°, panel (**D**); Supplementary Video S2). These chordae exert asymmetric traction during diastole, leading to incomplete cusp coaptation (X plane 0–90° mid-esophageal view, panel (**E**)) with type III aortic regurgitation according to the Carpentier classification, with a severe holodiastolic eccentric jet (VC 7 mm) directed posteriorly toward the AML (mid-esophageal 0°, panel (**F**); mid-esophageal 132°, panel (**G**); Supplementary Video S2). The occurrence of ACTS is rarely documented in the literature, and the genesis remains unclear. However, it has been hypothesized that they represent vestigial embryonic remnants arising during the morphogenesis of the aortic valve cusps [2]. This condition predominantly affects middle-aged Asian males with a tricuspid aortic valve morphology [3].

**Figure 3:**
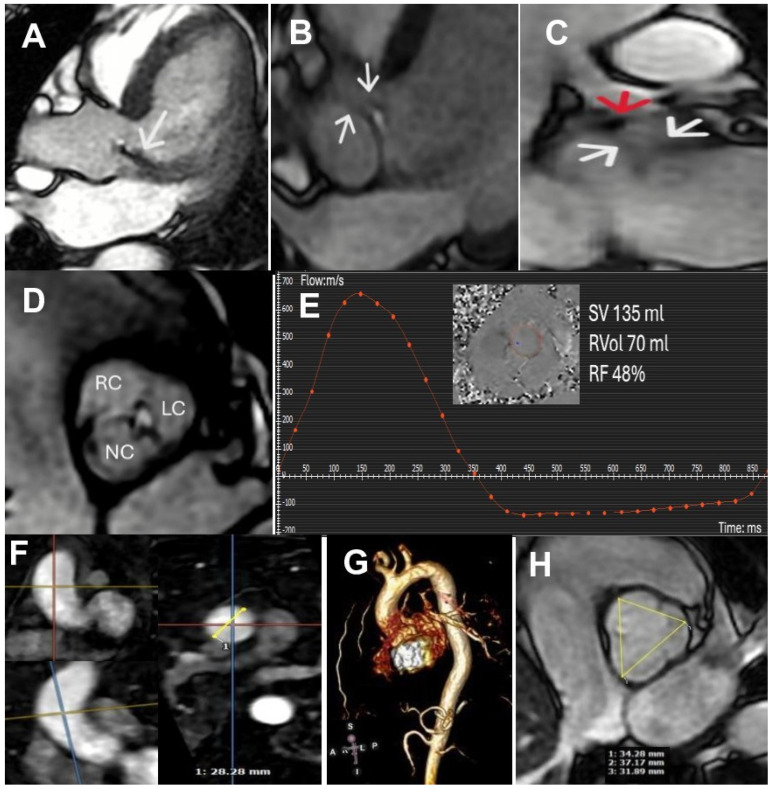
After TEE, aortic regurgitation was classified as severe, and the patient was referred for cardiac surgery, for which cardiac magnetic resonance imaging (cMRI) was performed to assess the thoracic aorta anatomy. Steady-state free precession (SSFP) cine imaging revealed in 3-chamber long-axis view in diastole an eccentric jet of aortic regurgitation visualized by the low signal due to spin-dephasing caused by shear and turbulence (white arrow, panel (**A**)). The spatial resolution of approximately 1–2 mm is insufficient to clearly visualize the ACTS; however, in the three-chamber long axis cine diastolic image (white arrows, panel (**B**)) and coronal cine diastolic image (white arrows, panel (**C**)), hypointense linear structures are observed, causing cusp retraction, and the high origin of the ACTS, at the level of the sinuses of Valsalva, is clearly visible as a focal nodular hypointensity (red arrow, panel (**C**)). SSFP short-axis cine image (panel (**D**); Supplementary Video S3) demonstrates asymmetric diastolic retraction of all three cusps, leading to incomplete leaflet coaptation. Finally, the biventricular function was quantified using short-axis SSFP cine images. Specifically, normal biventricular function was observed (LVEF 56%, RVEF 62%), along with moderate left ventricular dilation (EDVi 124 mL/m^2^). Contrast-enhanced MR angiography, utilizing multiplanar reconstructions, allowed for the assessment of the ascending aortic diameter, which was found to be within the normal range (double-oblique technique, panel (**F**); 3D aortic reconstruction, panel (**G**)). The aortic sinus-to-sinus diameter measurements fall within the normal range (SSFP cine short axis at aortic level, panel (**H**)) The quantification of aortic regurgitation was performed using through-plane phase contrast velocity mapping at the level of the ascending aorta. Moreover, cMRI confirmed severe aortic regurgitation, with a regurgitant volume (RVol) of 70 mL and a regurgitant fraction (RF) of 48% (panel (**E**)). Tissue characterization sequences, including late gadolinium enhancement and mapping, were negative. The patient, therefore, given the anatomical findings and young age, is awaiting surgical aortic valve replacement. cMRI proves to be a fundamental modality in the evaluation of valvular disease. It provides a comprehensive assessment by defining the regurgitation mechanism and quantifying RF and RVol, remaining the gold standard for biventricular function and volume analysis. Additionally, it enables detailed aortic anatomy assessment and myocardial tissue characterization, which has significant prognostic implications [4,5]. To date, this represents the first clinical case describing ACTS using cMRI, demonstrating the utility of this imaging technique in this setting. However, in our opinion, this case underscores the importance of a multimodality imaging approach. In fact, TTE remains the fundamental first-line modality, essential for initial disease characterization, identification of potential mechanisms, and severity assessment of regurgitation, guiding the need for further second-level imaging.

## Supplementary Materials

The following supporting information can be downloaded at: https://www.mdpi.com/article/10.3390/diagnostics15091071/s1, Video S1: TTE 2D cine parasternarnal long axis (PLAX) and short axis (PSAX) at great vessels level showing hyperechoic filaments (ACTS); Video S2: TEE 2D cine and 2D cine color mid-esophageal 130–148° showing ACTS causing aortic cusps tenting and regurgitation with an asymmetric jet toward AML; Video S3: cMRI bSSFP cine short axis view at aortic level showing cusps retraction and incomplete diastolic closure.
